# Hajdu–Cheney Syndrome in a Two-Generation Family: Longitudinal Skeletal Progression and Differential Therapeutic Responses in a Mother and Her Son

**DOI:** 10.3390/ijms27093788

**Published:** 2026-04-24

**Authors:** Ruggero Lanzafame, Thomas Zoller, Angelo Pietrobelli, Giorgio Piacentini, Rossella Gaudino, Alessandra Guzzo, Giovanni Adami, Francesco Pollastri, Franco Antoniazzi

**Affiliations:** 1 Pediatric Unit, Department of Surgical Science, Dentistry, Gynecology and Pediatrics, University of Verona, 37134 Verona, Italy; 2 Pennington Biomedical Research Center, Baton Rouge, LA 70808, USA; 3 Department of Pathology and Diagnostics, University of Verona, 37134 Verona, Italy; 4 Rheumatology Unit, Department of Medicine, University of Verona, 37134 Verona, Italy

**Keywords:** Hajdu–Cheney syndrome, *NOTCH2*, acro-osteolysis, osteoporosis, bisphosphonates, denosumab, vertebral fractures

## Abstract

Hajdu–Cheney syndrome (HCS) is a rare genetic skeletal disorder caused by truncating variants of *NOTCH2*, characterized by progressive bone resorption and marked phenotypic heterogeneity. Despite advances in understanding Notch signaling in skeletal biology, longitudinal clinical data tracking disease evolution from early childhood through adolescence are lacking. Here, we report a rare longitudinal intrafamilial observation of HCS in a mother and her son carrying the same *NOTCH2* pathogenic variant, providing novel insights into disease evolution and therapeutic response. Over extended follow-up, the son exhibited early vertebral fragility despite preserved or supranormal bone mineral density (BMD), whereas the mother developed severe osteoporosis, progressive acro-osteolysis, and multiple vertebral fractures. Longitudinal analysis revealed a dissociation between vertebral fragility and densitometric decline, challenging the paradigm that low BMD is the primary driver of skeletal morbidity in HCS. Treatment responses differed between the two patients, with bisphosphonate therapy in the son associated with stabilized BMD without altering vertebral structural progression, and denosumab in the mother associated with increased BMD, but not preventing progression of acro-osteolysis. Additionally, the emergence of extra-skeletal features during adolescence expands the phenotypic spectrum of HCS and suggests previously unrecognized systemic involvement. These data highlight intrinsic limitations of current therapeutic strategies and emphasize the need for targeted interventions addressing sustained Notch2 activation. Our findings contribute to the understanding of the natural history and therapeutic challenges of HCS, providing the framework for future mechanistic and translational research.

## 1. Introduction

Hajdu–Cheney syndrome (HCS, OMIM #102500) is a rare genetic disorder first described by Nicholas Hajdu in 1948 as a case of cranio-skeletal dysplasia, and later delineated as a syndrome by William Cheney in 1965. It arises from heterozygous truncating variants of the *NOTCH2* gene, resulting in a stabilized Notch2 protein and gain-of-function signaling. Epidemiologically, HCS has a prevalence of less than 1 in 1,000,000, with approximately 50–100 cases reported worldwide. It affects males and females equally, with no racial predisposition noted [1,2].

*NOTCH2* is part of the Notch family of transmembrane receptors (Notch1–4), which are activated by ligands such as Delta-like and Jagged proteins, leading to proteolytic cleavage and nuclear translocation of the intracellular domain to modulate gene expression. In HCS, truncating variants in the terminal exon 34 of *NOTCH2* eliminate the PEST (proline, glutamic acid, serine, threonine) domain, which is essential for ubiquitin-mediated degradation. This results in excessive Notch signaling due to protein stabilization [3]. The Notch pathway plays a critical role in osteoclastogenesis, osteoblast differentiation, and bone remodeling. Enhanced Notch2 activity promotes osteoclast maturation via NF-κB induction and RANKL upregulation, while inhibiting osteoblastogenesis, leading to the hallmark features of progressive bone resorption, fragility, and osteopenia [3]. Preclinical models, such as HCS mouse mutants with Notch2 variants mimicking human variants, exhibit osteopenia, increased osteoclast numbers, and elevated Rankl expression in osteoblasts and osteocytes, confirming the resorptive mechanism [3].

The main skeletal manifestations include progressive acro-osteolysis, severe osteoporosis with recurrent fractures, short stature and distinctive craniofacial anomalies, including micrognathia, hypertelorism, and wormian bones.

Extra-skeletal involvement further complicates management, including cardiovascular defects (e.g., patent ductus arteriosus, valvular defects, septal defects) in up to 10–20% of cases, renal abnormalities (e.g., polycystic kidneys), hearing loss, and occasional splenomegaly. Neurological complications arise from basilar invagination or syringomyelia, potentially leading to hydrocephalus or neural compression. Dental anomalies, such as premature tooth loss and root resorption, are common due to alveolar bone involvement [1,2,4].

Despite advances in understanding the molecular basis of HCS, its natural history from early childhood through adolescence remains poorly defined. Most available clinical data derive from cross-sectional adult cohorts or isolated pediatric reports, limiting insight into early skeletal evolution and age-dependent disease progression. Moreover, although antiresorptive therapies such as bisphosphonates [2,5] and denosumab [6] have been used empirically, their long-term impact on vertebral integrity and structural disease progression remains unclear.

Familial cases of HCS provide a unique opportunity to explore phenotypic variability and treatment response within a shared genetic background. However, longitudinal intrafamilial comparisons spanning pediatric and adult disease stages are extremely rare.

In this context, we report a two-generation family carrying a truncating *NOTCH2* variant, representing a rare longitudinal observation of skeletal evolution from infancy through adolescence. By describing the clinical course of a severely affected mother treated with denosumab (previously described by Adami et al. [6]) and her son treated with bisphosphonates, this study provides novel insights into disease progression, dissociation between bone mineral density and skeletal fragility, and the limitations of current therapeutic strategies in *NOTCH2*-driven disease.

## 2. Case Reports

The mother’s presentation exemplifies the severe end of the HCS spectrum. At age 33, she presented with generalized bone pain, particularly in the hands, hips, and back, alongside a history of multiple vertebral fractures from T6 to L3 and non-vertebral fractures (left knee, right tibia, fibula, and 4th metatarsal) over 14 years [6]. She reported progressive shortening of fingers, consistent with acro-osteolysis. Physical examination revealed short stature (155 cm), prominent forehead, hypertelorism, micrognathia, stubby hands with finger deformities, hallux valgus, and palpable spleen. No neurological, cardiovascular, or renal symptoms were noted, though abdominal ultrasound confirmed splenomegaly and no polycystic kidneys [6].

**Figure 1:**
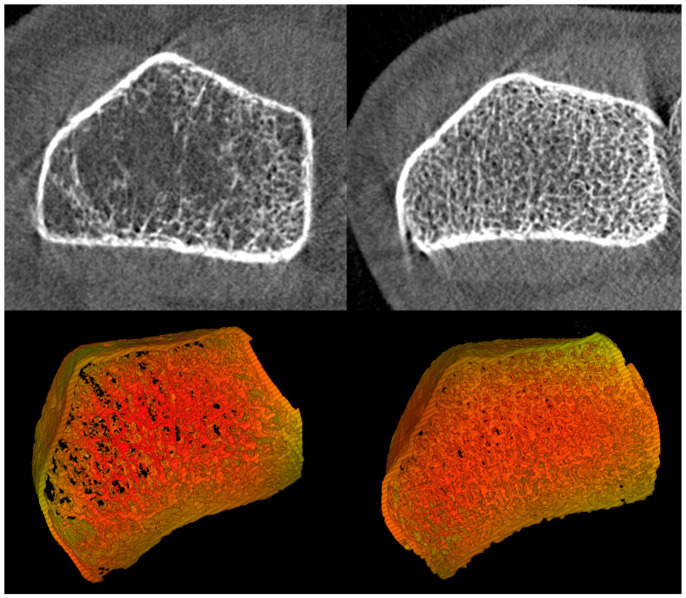
High-resolution peripheral quantitative computed tomography (HR-pQCT) images of the distal phalanx. Left: mother; right: age- and sex-matched control. Upper panels show axial grayscale images, while lower panels display corresponding three-dimensional reconstructions. The mother exhibits a marked reduction in total bone volume compared with the control, with relative preservation of cortical and trabecular thickness.

**Figure 2:**
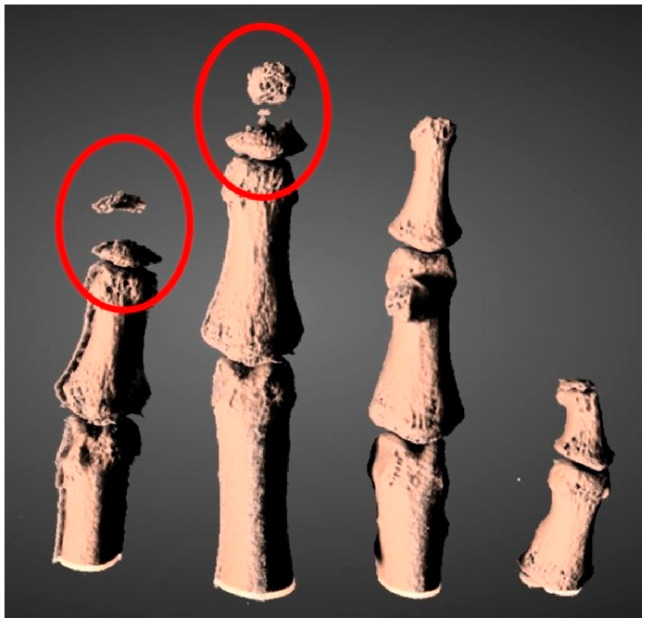
Three-dimensional HR-pQCT reconstruction of the distal phalanges showing severe acro-osteolysis, with marked resorption and fragmentation of the distal tufts (highlighted).

Laboratory evaluations were normal for complete blood count, electrolytes, liver and renal function. Bone turnover markers (serum CTX, BSAP, P1NP) were within the expected range, except for elevated RANKL (0.451 pmol/L) and reduced OPG (1.244 pmol/L), suggesting an imbalance favoring osteoclast activation and increased bone resorption. Dual-energy X-ray Absorptiometry (DXA) showed lumbar spine bone mineral density (BMD) 0.810 g/cm^2^ (T-score −3.1), femoral neck 0.848 g/cm^2^ (T-score −1.4), and total hip 0.870 g/cm^2^ (T-score −1.1). Imaging confirmed vertebral deformities, scoliosis, skull anomalies (increased anteroposterior diameter, paranasal sinus hypoplasia, open lambdoid suture), hand acro-osteolysis (2nd, 3rd and 5th fingers, Figure 1 and Figure 2), and foot demineralization with deformities. Genetic sequencing revealed a heterozygous nonsense variant in exon 34 (c.6667C>T; p.Gln2223Ter) of *NOTCH2*, creating Gln2223Ter, a nonsense variant upstream of the PEST domain, consistent with gain-of-function. Variant detection was performed using next-generation sequencing, and the heterozygous state was confirmed by visualization of sequencing reads showing balanced allele representation.

Several treatments were previously administered with limited clinical benefit, including vitamin D, pamidronate (once in 2008), clodronate (twice in 2009), and strontium ranelate (five months in 2010). Denosumab 60 mg subcutaneously every 6 months was initiated in October 2013, along with vitamin D3 10,000 IU weekly. Serum RANKL dropped post-dose, CTX suppressed initially but normalized pre-subsequent doses, and bone formation markers remained stable. BMD increased by 6.8% at the lumbar spine and 2.6% at femoral neck over 2 years, with no new fractures (verified by Vertebral Fracture Assessment—VFA, X-rays and Magnetic Resonance Imaging—MRI).

Unfortunately, after the case report by Adami et al. [6], the patient was lost to follow-up for several years and subsequently re-presented with an ankle fracture, which occurred after temporary discontinuation of denosumab therapy, recommended elsewhere for left total hip arthroplasty due to osteoarthritis. Approximately six months after the fracture, upon resumption of denosumab, biochemical parameters returned to baseline values (CTX 0.294, PTH 43 pg/mL, serum calcium 2.33 mmol/L, phosphate 1.11 mmol/L, 25OH-vitamin D 57.3 ng/mL) and remained stable through December 2025, the time of the last follow-up visit.

This stability was further confirmed both by the absence of fractures and from a densitometric perspective, with DXA scans from 2022 to 2025 showing stable values: lumbar spine BMD from 0.741 to 0.745 g/cm^2^ (last T-score −3.5), femoral neck BMD from 0.741 to 0.745 g/cm^2^ (last T-score −1.8), and total hip BMD from 0.776 to 0.806 g/cm^2^ (last T-score −1.6).

In this context, to obtain a more detailed assessment, a high-resolution peripheral quantitative computed tomography (HR-pQCT) scan was also performed. HR-pQCT scans demonstrated that total bone volume was markedly reduced in the mother compared with an age- and sex-matched control, whereas cortical and trabecular thickness appeared relatively preserved (Figure 1). Although baseline HR-pQCT data were not available, it can be hypothesized that denosumab therapy may have normalized the reversible components of bone (i.e., cortical and trabecular thickness) without modifying the underlying pathological bone architecture.

**Figure 3:**
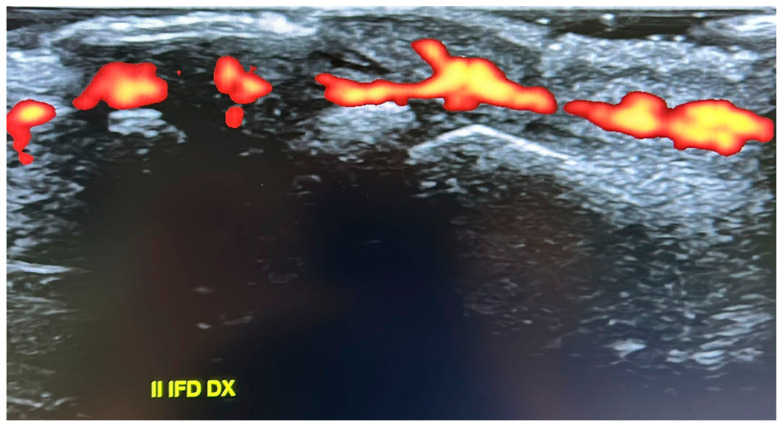
Ultrasound evaluation of the interphalangeal joints demonstrating a marked inflammatory signal on power Doppler imaging, consistent with active synovial inflammation.

Despite this metabolic stability, the patient reported worsening pain, particularly at the level of the second distal interphalangeal joint of the right hand. In this context, HR-pQCT revealed severe acro-osteolysis (Figure 2), and ultrasound evaluation demonstrated a marked inflammatory signal (Figure 3). Given evidence suggesting that NOTCH2 signaling may promote NF-κB activation and downstream transcriptional pathways [7], treatment with an anti-TNF agent was initiated (adalimumab initially, discontinued due to a cutaneous reaction, followed by golimumab). This therapy was maintained for approximately 18 months and was associated with a significant reduction in pain and complete resolution of the power Doppler signal. Treatment was subsequently discontinued (August 2022 to May 2024), with stability confirmed at follow-up visits. Consistent findings were observed in HR-pQCT measurements of the distal interphalangeal joint, with total bone volume remaining stable over the 18-month period from initiation to discontinuation of anti-TNF therapy.

**Figure 4:**
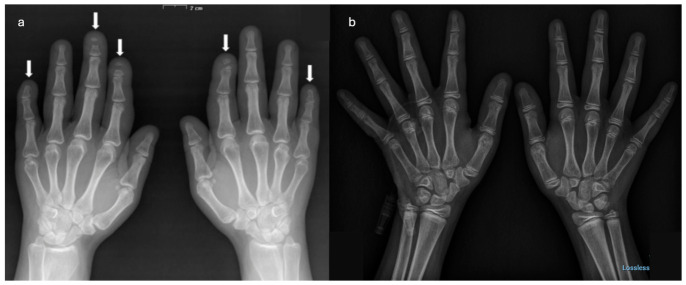
Comparative hand radiographs of the mother (**a**) and son (**b**). Anteroposterior views show advanced distal phalangeal resorption in the mother as highlighted by the arrows (**a**) versus absent acro-osteolysis and preserved metacarpal and joint integrity in the son (**b**).

Her son, born at 37 weeks (2910 g, 46 cm), required neonatal respiratory support for distress but had normal early development except for RSV bronchiolitis at 6 months. No chronic respiratory sequelae were noted. He experienced normal neurocognitive development and engaged in regular physical activities, including karate and swimming. At age five, a low-trauma clavicle fracture prompted evaluation. Height and weight were normal (25th percentile), with Tanner stage A1/P1/G1. Physical examination showed micrognathia but no other dysmorphic features. A DXA was performed with a QDR Hologic Delphy, which revealed a lumbar spine (L1–L4) BMD of 0.633 g/cm^2^ and Z-score −0.1, and whole left femur BMD was 0.832 g/cm^2^ and Z-score +1.7. He presented in good condition with normal anthropometrics values, and X-ray performed on both hands showed no acro-osteolysis (Figure 4b). Targeted sequencing of the *NOTCH2* gene confirmed the same heterozygous nonsense variant in exon 34 (c.6667C>T; p.Gln2223Ter) of his mother. Treatment with vitamin D and Calcium supplementation was started. The subsequent five years of follow-up were uneventful.

**Figure 5:**
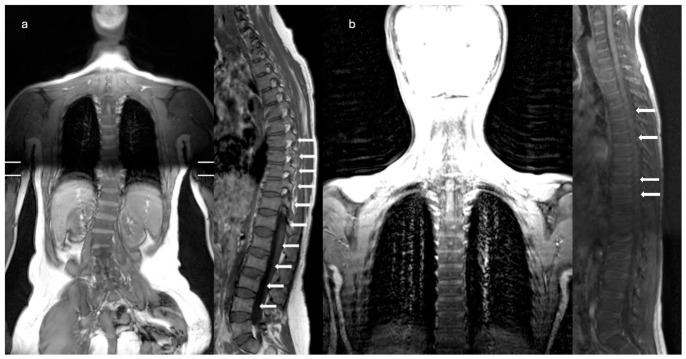
Comparative vertebral MRI scans of the mother (**a**) and son (**b**). Coronal T1/T2-weighted sequences highlight thoraco-lumbar scoliosis in the mother (**a**), versus no scoliosis in the son. Sagittal T1/T2-weighted sequences reveal chronic deformities with marrow changes in the mother as highlighted by the arrows (**a**), versus early biconcave fractures as highlighted by the arrows at T4, T6, T9, and T10 in the son, without canal compromise (**b**).

By age 11, he started complaining of back pain which was worsening with movement. A magnetic resonance imaging (MRI) of the spine showed multiple vertebral fractures in D4, D6, D9, and D10 (Figure 5b). DXA imaging was repeated, revealing a worsening BMD at the lumbar spine: lumbar spine (L1–L4) showed a Z-score of −1.2, while whole left femur Z-score of +1.1. As a consequence of vertebral fracture and progressive instability of the spine, a supporting brace was prescribed, and bisphosphonates treatment with Neridronate was started with a dose of 2 mg/kg every three months, along with continuation of vitamin D and calcium supplementation.

**Figure 6:**
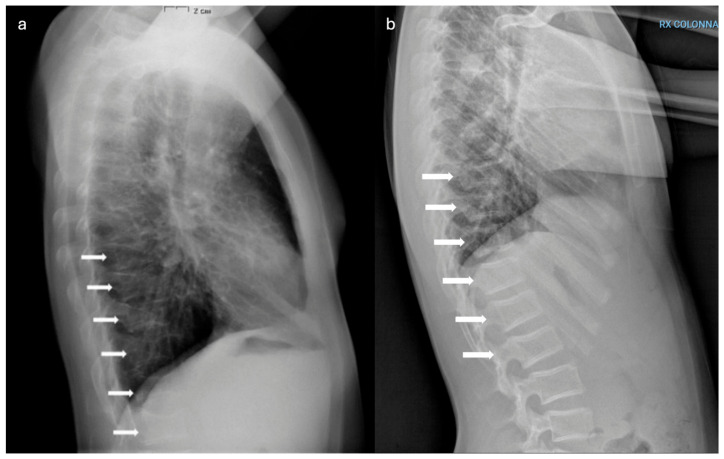
Comparative lateral spine radiographs of the mother (**a**) and son (**b**). Images demonstrate multiple compression fractures as highlighted by the arrows, and kyphosis in the mother (**a**), compared with mild endplate deformities at T9–T12 in the son and early superior endplate deformities in L1–L2, as highlighted by the arrows (**b**).

The patient then sustained a fracture of the 5th right metatarsal neck and proximal phalanx of the right 5th finger at the age of 12 years old. A spine X-ray at that time also revealed reduced vertebral height at D9–D12; early superior endplate deformities in L1–L2 (Figure 6b). He then sustained a subsequent fracture of the left great toe at the age of 14 years old.

Growth and puberty progressed regularly. At the age of 14 years old his height was 160.4 cm (–1 SDS), weight 65 kg (+1.6 SDS), BMI 24.9 kg/m^2^. Tanner stage: A2; P2; testicular volume 12–13 mL. Abdominal ultrasound revealed hepatic steatosis with hepatomegaly and splenomegaly (longitudinal axis 13 cm); normal biliary, renal, and pancreatic anatomy. Routine hematologic and biochemical parameters remained within normal ranges, including hepatic and renal function, glucose, cholesterol and triglycerides; normal blood count, vitamin D levels and serum calcium, phosphate, ALP and PTH. Urine calcium and phosphate excretion remained within normal limits.

He is now 15 years old and clinically well, with a diet adequate in calcium, stable laboratory parameters, and no new fractures in the last year. His last DXA showed a lumbar spine (L1–L4) BMD of 0.861–0.998 g/cm^2^ with Z-scores −1.0 to −0.2 (total −0.6); femur BMD 1.093–1.376 g/cm^2^ with Z-scores +0.9 to +2.2 (total +1.9). He is still receiving Neridronate infusions of 2 mg/kg every three months and a daily vitamin D intake of 1000 IU.

Comparative imaging (Figure 4, Figure 5 and Figure 6) highlights intrafamilial variability in skeletal involvement: mother’s advanced phalangeal resorption (Figure 4a) vs. son’s normal hand radiographs (Figure 4b); chronic vertebral deformities and scoliosis in mother (Figure 5a) vs. early biconcave fractures in son (Figure 5b); multiple compression fractures and kyphosis in mother (Figure 6a) vs. multiple mild endplate deformities in son (Figure 6b).

## 3. Discussion

This familial case demonstrates several key features of HCS and its intrafamilial variability. Despite carrying the same *NOTCH2* pathogenic variant, the previously described mother and her son displayed markedly different disease severity. However, the comparison between the mother and son is inherently limited by substantial differences in age, sex, developmental stage, and disease duration. Therefore, the observed variability should be interpreted cautiously and cannot be attributed solely to genetic or biological modifiers within the same family. Modifying factors may include hormonal influences, lifestyle differences, age-dependent progression or stochastic variation in Notch signaling.

Early vertebral involvement despite relatively preserved BMD (with femoral values remaining above average and lumbar Z-scores not falling below −1.2) suggests that skeletal fragility in HCS may not be fully captured by densitometric measurements alone. To our knowledge, this represents one of the few longitudinal clinical observations in HCS documenting vertebral fragility preceding densitometric deterioration. Abnormal osteoclast and osteoblast dynamics, rather than mineral density, likely drive fragility as seen in mouse models with increased osteoclastogenesis and Rankl expression [2,3].

In the present report, Neridronate stabilized BMD and slightly reduced fracture rates in the son, but did not arrest vertebral changes. Bisphosphonates like pamidronate and zoledronic acid have also shown variable BMD gains but no impact on acro-osteolysis, likely because they suppress osteoclasts without addressing upstream Notch-driven RANKL upregulation [8,9,10]. Denosumab resulted in significant BMD gains in the mother (6.8% lumbar, 2.6% femoral neck), but did not prevent progression of acro-osteolysis; this further supports the concept that suppression of RANKL-mediated osteoclast activity is insufficient to counteract sustained Notch2 signaling. Denosumab also carries the intrinsic risk of rebound bone loss and hypercalcemia. Elevated RANKL and low OPG in the mother support enhanced osteoclast activity, and denosumab’s suppression of RANKL explains the response, though acro-osteolysis progressed, suggesting that additional local mechanisms (e.g., neovascularization or inflammation) may be involved, independent of systemic resorption [6,11]. These observations should be interpreted descriptively, as the two described patients differed substantially in age, disease stage, prior treatments, and clinical context. Therefore, no direct comparison of therapeutic efficacy can be drawn. Rather, the data suggest that currently available antiresorptive therapies may improve − or at least stabilize−densitometric parameters without fully modifying the structural progression of HCS.

The identification of hepatic steatosis and splenomegaly during adolescence may further expand the phenotypic spectrum of HCS. While Notch signaling has been implicated in metabolic regulation and immune cell differentiation, the relationship between *NOTCH2* gain-of-function and these findings remains unclear. Alternative explanations, including metabolic factors related to body composition or incidental findings, should also be considered. However, given the known role of Notch signaling in hepatic lipid metabolism and splenic marginal zone development [4], these observations could suggest a broader systemic involvement and warrant further investigation in larger cohorts. Comprehensive monitoring beyond skeletal assessments is warranted, including ultrasound for renal/splenic anomalies and echocardiography for cardiac defects.

Phenotypic heterogeneity in HCS is well-documented, with age-dependent presentations: craniofacial features and wormian bones in infancy, acro-osteolysis and short stature in childhood, progressive osteoporosis and fractures in adulthood. Inter- and intra-familial variability, as described in our report, suggest modifiers like sex hormones or epigenetics. Genetic studies confirm all HCS variants cluster in exon 34 of *NOTCH2*, causing PEST loss and gain-of-function [1,2,8,9]. Somatic *NOTCH2* variants in B-cell lymphomas highlight oncogenic potential, but malignancy risk in HCS remains low [2,9].

Diagnostic challenges arise from overlapping syndromes (e.g., serpentine fibula-polycystic kidney syndrome, now recognized as HCS variant [9]). Brennan and Pauli’s criteria aid diagnosis of HCS: Wormian bones or open sutures, platybasia, premature loss of teeth, micrognathia, coarse face, coarse hair, midfacial flattening and short stature (<5th percentile). The clinical diagnosis is established when acro-osteolysis plus three manifestations are found in adults, or four manifestations excluding acro-osteolysis in children; patients with documented family history only need two manifestations for the clinical diagnosis [9]. Genetic confirmation is essential, with sequencing of *NOTCH2* exon 34 [9,10,11].

Therapeutic gaps persist; antiresorptives provide partial benefits [2,5,6,10], but novel treatments like targeted Notch inhibitors (e.g., gamma-secretase inhibitors) are needed. Preclinical data support RANKL blockade, but clinical trials are lacking due to the rarity of the condition. Anti-TNF therapy proved effective during phases of inflammatory exacerbation, leading to both clinical improvement and resolution of the ultrasound inflammatory signal. This suggests that it may represent a valuable adjunctive therapeutic option to be considered during disease flares; however, additional data are needed to support its routine use in this setting. Multidisciplinary care—orthopedics for fractures, dentistry, neurology for basilar invagination—is crucial.

The use of highly sensitive diagnostic techniques plays a pivotal role in these complex and uncommon scenarios, allowing for a more accurate characterization of disease activity and treatment response; in this context, HR-pQCT emerged as a valuable tool in the longitudinal assessment of this rare condition, providing microarchitectural and functional insights that extend beyond conventional densitometry. Specifically, it allows a more comprehensive evaluation of treatment effects by capturing aspects of bone structure and volumetric parameters that are not reflected by BMD or standard radiography.

This study has several limitations. First, it is based on a very small sample size (two related individuals), which limits the generalizability of the findings. Second, the observational nature of the report precludes any causal inference regarding disease mechanisms or treatment effects. In addition, the comparison between the two patients is inherently confounded by differences in age, sex, developmental stage, disease duration, and prior treatments. Therefore, therapeutic responses should be interpreted descriptively rather than comparatively. These limitations should be considered when interpreting the findings.

## 4. Conclusions

This two-generation report represents a rare longitudinal, intrafamilial characterization of HCS across different stages of life. Our findings suggest that vertebral fragility may occur even in the presence of non-severely reduced BMD, indicating that densitometric measurements alone may not fully capture skeletal risk in NOTCH2-related disease.

Treatment responses observed in this family highlight the potential for partial benefits of antiresorptive therapies on BMD, while also underscoring their limited impact on structural disease progression. Emerging therapies, such as anti-TNF agents, show a promising rationale during disease flares; however, robust clinical data are still lacking.

Overall, these observations contribute to a better understanding of HCS and underscore the need for further studies to clarify disease mechanisms and optimize therapeutic strategies.

## Data Availability

The data regarding the clinical case presented in this study are not publicly available due to privacy and ethical restrictions.

## Abbreviations

The following abbreviations are used in this manuscript:

BMD	Bone Mineral Density
BSAP	Bone-Specific Alkaline Phosphatase
CTX	C-terminal telopeptide
DXA	Dual-energy X-ray Absorptiometry
HCS	Hajdu–Cheney syndrome
HR-pQCT	High-Resolution Peripheral Quantitative Computed Tomography
MRI	Magnetic Resonance Imaging
P1NP	Procollagen type 1 N-terminal propeptide
